# Surface Nanocrystallization and Amorphization of Dual-Phase TC11 Titanium Alloys under Laser Induced Ultrahigh Strain-Rate Plastic Deformation

**DOI:** 10.3390/ma11040563

**Published:** 2018-04-06

**Authors:** Sihai Luo, Liucheng Zhou, Xuede Wang, Xin Cao, Xiangfan Nie, Weifeng He

**Affiliations:** 1 Science and Technology on Plasma Dynamics Laboratory, Air Force Engineering University, Xi’an 710038, China; luo_hai@126.com (S.L.); happyzlch@163.com (L.Z.); wangxuede@163.com (X.W.); studentcaoxin@163.com (X.C.); 2 Institute of Aeronautics Engine, School of Mechanical Engineering, Xi’an Jiaotong University, Xi’an 710049, China; 3 School of Mechanical and Power Engineering, East China University of Science and Technology, Shanghai 200237, China

**Keywords:** laser shock peening, dual-phase TC11 titanium alloy, ultrahigh strain-rate plastic deformation, nanocrystallization, amorphization

## Abstract

As an innovative surface technology for ultrahigh strain-rate plastic deformation, laser shock peening (LSP) was applied to the dual-phase TC11 titanium alloy to fabricate an amorphous and nanocrystalline surface layer at room temperature. X-ray diffraction, transmission electron microscopy, and high-resolution transmission electron microscopy (HRTEM) were used to investigate the microstructural evolution, and the deformation mechanism was discussed. The results showed that a surface nanostructured surface layer was synthesized after LSP treatment with adequate laser parameters. Simultaneously, the behavior of dislocations was also studied for different laser parameters. The rapid slipping, accumulation, annihilation, and rearrangement of dislocations under the laser-induced shock waves contributed greatly to the surface nanocrystallization. In addition, a 10 nm-thick amorphous structure layer was found through HRTEM in the top surface and the formation mechanism was attributed to the local temperature rising to the melting point, followed by its subsequent fast cooling.

## 1. Introduction

Titanium alloys are the most widely utilized alloy in the aero-engine industry. Due to their high fatigue strength, low density, and high corrosion resistance, they are employed in components such as disks, fans, and compressor blades. Nonetheless, the fatigue failure of titanium alloy blades, especially those subjected to foreign object damage, has been a major concern [1,2]. In order to improve the fatigue resistance, various surface modification techniques, such as mechanical attrition treatment (SMAT), high-pressure torsion, and shot peening, have been proposed to improve the mechanical properties and fatigue strength of titanium alloys [2,3,4]. One mechanism of fatigue strength improvement is to induce surface nanocrystallization, the main reason is that nanocrystalline materials have many exceptional physical, mechanical, and fatigue resistance properties, relative to their coarse-grained counterparts [5,6,7,8,9,10]. However, the drawback of these proposed processing techniques is that they are only suitable for the simple components, which restricted their industrial application to aero-engine blades.

Compared with other surface treatment technologies, laser shock peening (LSP) have the remarkable advantages of not requiring contact with the substrate, of affecting a deep layer, and of having an excellent controllability, which makes LSP very suitable for complex structures, for example, aero-engine blades [11,12,13]. Similar to conventional shot peening, LSP is capable of refining grains and producing a surface nanostructured layer [14,15,16,17,18,19,20,21,22,23,24,25,26]. Recently, LSP has been used on the aero-engine components to improve the high cycle fatigue performance and to induce surface nanocrystallization of stainless steels [18,19], titanium alloys [20,21,22], and nickel-based superalloys [23,24] has been reported. Lu et al. [14,15,16] discussed, in detail, the mechanism of grain refinement induced by LSP on LY2 aluminum alloy, AISI 304 stainless steel, and commercially pure titanium. Luo et al. [25] further analyzed the mechanism of surface nanocrystallization induced by LSP on the metallic alloys with different stacking fault energies. Moreover, Lainé et al. [26] analyzed, in detail, the effects of metallic shot peening (MSP) and LSP on the microstructure of Ti-6Al-4V. The results showed that the grain refinement of MSP was the evolution of tangled dislocation structures and shear bands, whereas that of LSP was the evolution of directional planar dislocations and networks of dislocation cells and sub-grains. In addition, the formation of an amorphous structure was noticed on the NiTi shape memory alloy and silicon after laser-induced shock wave compression [27,28]. To summarize, LSP is beneficial to the microstructural change of the surface layer and thus, improves the fatigue strength. Thus, it is of great interest to investigate the microstructural evolution mechanism of the TC11 titanium alloy under LSP treatment.

In this work, the microstructures of dual-phase TC11 titanium alloys treated by LSP were characterized by transmission electron microscopy (TEM). The microstructural evolution rule and surface nanocrystallization mechanism were investigated. In addition, a special phenomenon of surface amorphization on the top layer was observed by high-resolution transmission electron microscopy (HRTEM), and the formation mechanism was also discussed.

## 2. Experimental Procedures

### 2.1. Materials

**Figure 1:**
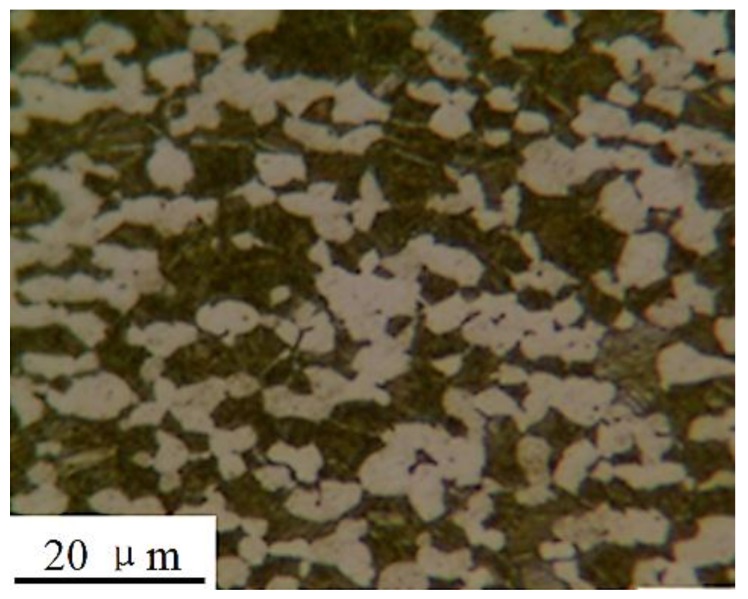
An optical micrograph of the TC11 titanium alloy (etched for ~12 s in a solution of 5% HNO_3_ and 95% of absolute alcohol).

TC11 titanium alloy is typically employed for fan blades in the Chinese aero-engine industry. The chemical composition (in wt %) is given in Table 1. The sample was an α + β type heat-resistant titanium alloy, composed of a primary hexagonal close-packed (hcp) α phase and a lamellar transformed bcc β phase. The equiaxial α-grains with an average size of 10 μm and the acicular β-grains were observed by the optical microscopy, as shown in Figure 1. The alloy was subjected to a double annealing heat treatment for 2 h at 950–980 °C followed by air cooling, and heating for 6 h at 530 °C, followed by 6 h of air cooling. The basic mechanical properties of these titanium alloys are shown in Table 2.

### 2.2. Principle and Experimental Procedure of LSP

**Figure 2:**
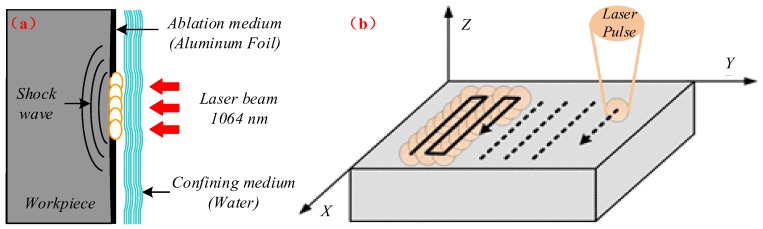
Schematic illustrations of the laser shock peening process. (**a**) The plasma shock wave generated by nanosecond pulse laser; (**b**) The LSP processed area of samples for microstructural observation and laser shock paths (the LSP processed area had the dimensions of 25 × 20 mm).

In the LSP process, a laser pulse with a short pulse width (ns) and a high power density (GW/cm^2^) was placed on the workpiece surface. The workpiece to be laser peened was covered by two different layers, namely an opaque ablating layer (Al foil/black paint) and a transparent confining layer (water/glass), as shown in Figure 2. The laser pulse passed through the transparent confining medium and struck the ablating layer. It was then absorbed by the ablating layer, which immediately vaporized and formed plasmas of a high temperature and pressure. The expansion of the plasma detonation wave led to the formation of a shock wave that propagated into the target with an intensity of several GPa. When the shockwave pressure was larger than the material dynamic yield strength, a plastic deformation was produced and resulted in the generation of compressive residual stresses and microstructural changes in the material surface layer.

Before LSP treatment, the sample surface was polished with SiC paper with a grit number ranging between 400 and 2000. An ultrasound ethanol bath was then used to clean the surface of the sample. During the LSP experiment, the confining layer and ablating layers consisted of floating water with a thickness of about 1–2 mm and a 0.1 mm-thick Al foil, respectively. The titanium alloys were machined into square samples (40 mm × 40 mm × 4 mm) which were mounted on a five-coordinate robot arm. The robot arm was controlled to move in the x-y direction in order to achieve the designed laser paths, shown in Figure 2. The laser pulse with a wavelength of 1064 nm and a pulse of around 20 ns was generated by a Q-switched self-designed Nd:YAG laser (SGR-EXTRA/25J). The laser spot diameter, overlapping-rate, and repletion-rate were 3 mm, 50%, and 1 Hz, respectively. In order to investigate the effect of laser parameters on the microstructural characteristics, different laser power densities (2.83, 4.24, and 5.66 GW/cm^2^) at three impacts were adopted.

### 2.3. Microstructural Observations

X-ray diffraction (XRD) analyses of the TC11 titanium alloy with and without LSP treatment were conducted using an MFS-7000 X-ray diffractometer with Cu-Kα radiation (Shimadzu, Kyoto, Japan). The take-off angle was 6° and the generator settings were 40 kV and 35 mA. The diffraction data were collected for values of 2*θ* ranging from 30° to 80° at a step of 0.02° and a time step of 5 s. TEM HRTEM observations on the LSP-treated samples were performed using a JEM-2100F (Japan Electron Optics Laboratory, Beijing, China) with the following experimental parameters: FEG (field emission gun): 200 kV; point resolution: 0.23 nm; and line resolution: 0.14 nm. The TEM foils for the surface layers of the samples were prepared by mechanically grinding the samples on the side that had not been subjected to LSP in order to obtain thin plates with a thickness of about 50 μm. The thin plates were then electro-polished using a twin-jet technique in a liquid solution consisting of 300 mL of CH_3_OH, 175 mL of C_4_H_9_OH, and 30 mL of HClO_4_ (30% solubility). The grain size measurements were made directly from the dark-field TEM images. In addition, the focused ion beam (FIB) lift-out method was used to prepare the cross-sectional TEM samples from the top surface of the LSP-treated sample in the FEI Helios NanoLab^TM^ 600i system, and the HRTEM observation was used to analyze the microstructural characteristics at different depths.

## 3. Results and Discussion

### 3.1. Microstructure Characterization on the Surface

**Figure 3:**
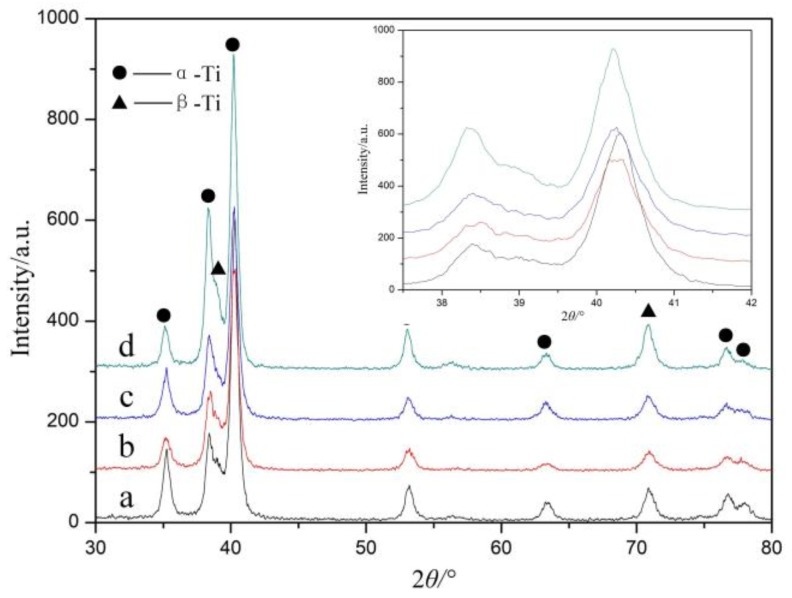
The X-ray diffraction pattern of the surface microstructure with different laser power density at three impacts. (**a**) original; (**b**) 2.83 GW/cm^2^; (**c**) 4.24 GW/cm^2^; (**d**) 5.66 GW/cm^2^. The inset shows the XRD pattern for 2*θ* from 37.5° to 42°.

Figure 3 shows the XRD patterns of the LSP-treated specimens with different laser power densities at three impacts. After LSP treatment, the Bragg diffraction peak of the TC11 titanium alloy became broader and decreased in intensity, which indicated that the grain refinement, lattice deformation, and micro-strain increases had been induced on the surface layer of the alloy. It is worth noting that, as the power density increased, the Bragg diffraction peaks broadened more significantly and eventually flattened out. On the other hand, the XRD spectral peak and position remained virtually unchanged, indicating that no phase change had occurred. The ultrahigh strain rate plastic deformation was induced during the LSP process, which resulted in the generation of a non-uniform residual elastic micro-strain in the materials and a microstructural change. This was the reason for the tendency of the broadening of the diffraction peak to deviate towards low angles, as seen in the inset of Figure 3.

The XRD patterns showed that the laser power density had a direct influence on the microstructural change following LSP treatment. The XRD measurement results were relative to the surface layer with a depth of about 1 μm (the penetrated depth of the X-ray). Thus, in order to further confirm the grain refinement induced by the LSP treatment and to analyze the microstructural evolution of the TC11 titanium alloy under ultrahigh strain-rate deformation, TEM observations were carried out.

**Figure 4:**
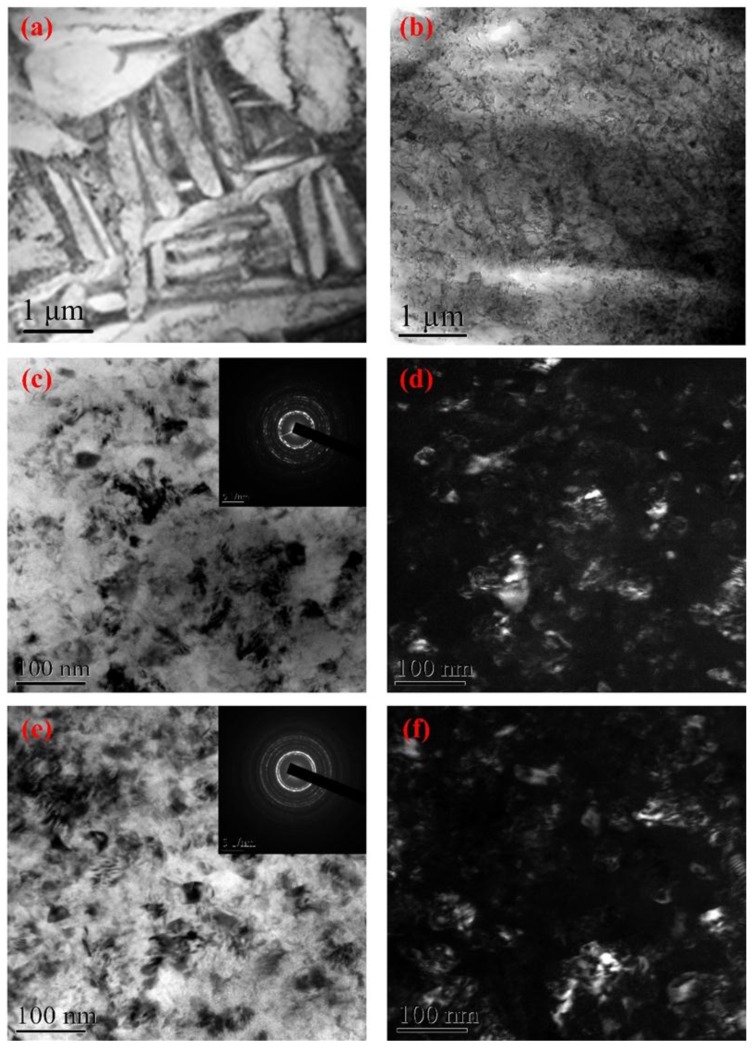
The transmission electron microscopy TEM images and corresponding diffraction patterns of the surface layer of the TC11 titanium alloy samples after LSP with different laser power densities at three impacts. (**a**) without LSP; (**b**) 2.83 GW/cm^2^; (**c**) 4.24 GW/cm^2^, the inset is the corresponding SAED pattern; (**d**) the corresponding dark-field image of (c); (**e**) 5.66 GW/cm^2^, the inset is the corresponding SAED pattern; (**f**) the corresponding dark-field of (e).

Figure 4 shows the TEM images obtained from the top surface layer after LSP treatment. The original TC11 titanium alloy was composed of α and β phases and the phase boundary, as seen in Figure 4a. The greatest dimension of these phases reached several micrometers. After LSP with a low laser power density (2.83 GW/cm^2^), high-density dislocation configurations (dislocation, dislocation tangle, and dislocation cell) were generated near the grain boundaries, as shown in Figure 4b. When the laser power density increased to 4.24 GW/cm^2^, many nanocrystalline artifacts were generated, as shown in Figure 4c,d. The corresponding selected area electron diffraction (SAED) pattern was dominated by circles, which indicated the random orientations of the nanocrystalline artifacts and their high angle grain boundaries. When the power density was increased to 5.66 GW/cm^2^, the surface nanocrystallization was completed, as shown in Figure 4e,f, and the grain size was refiner and more uniform compared to that induced by lower laser power densities. The corresponding SAED pattern presented continuous, homogeneous and broadened concentric rings, confirming the random crystallographic orientation of the grains. In a previous work, we investigated the effect of multiple LSP impacts on the TC11 titanium alloy at a power density of 4.24 GW/cm^2^ [29] and found that the high-density dislocations and dislocation walls were formed after one impact, and the numerous nanocrystalline artifacts were generated after three LSP impacts. When the number of LSP impacts increased to five, the average grain size decreased to about 40 nm and the grain orientation of the nanocrystalline artifacts became more random and uniform. Therefore, increasing either the laser power density or the LSP impacts was effective in inducing surface nanocrystallization. In other words, grain refinement increases with the amount of laser energy injected into the materials.

**Figure 5:**
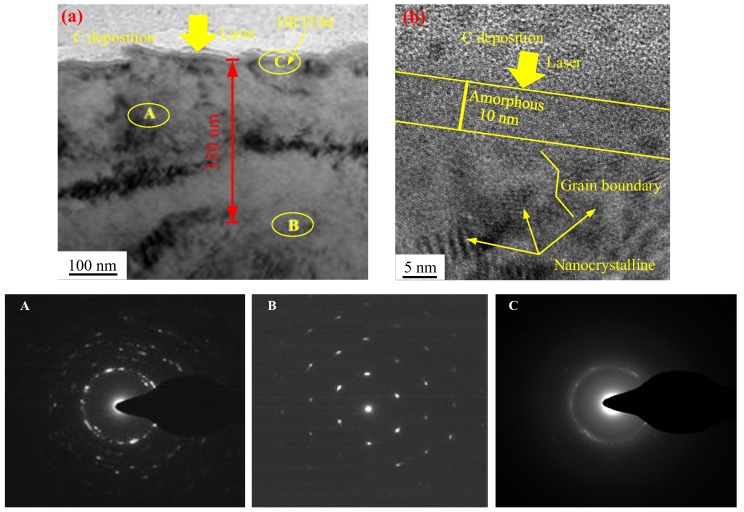
TEM photographs and selected diffraction patterns in cross-section and HRTEM photographs on the top surface layer of TC11 titanium alloy treated by LSP with three impacts. (**a**) The cross-sectional TEM image, and the plastic deformation layer is divided into three layers, typically regions, A, B and C). (**b**) The HRTEM observation of region C in (**a**).

To investigate the microstructural characteristics at different depths of TC11 titanium alloys after LSP treatment. The chosen sample was the one that had been subjected to LSP with a power density of 4.24 GW/cm^2^ at three impacts. The cross-sectional microstructure of TC11 titanium alloy is shown in Figure 5. The top region in Figure 5a was a carbon (C) deposition layer which was used to protect the sample from the ion beam during the TEM sample preparation by FIB. The integrity of the C deposition layer showed that the sample had not been damaged during the preparation process. The SAED pattern in the selected region, A, showed that the nanostructure was produced after LSP treatment, consistent with the results reported in Figure 5. Beneath the nanocrystalline layer, at a depth of about 350 nm, the slight elongation of the corresponding SAED pattern points indicated to the presence of high-density dislocations and sub-structures in the selected region, B. The microstructural characteristics at different depths were consistent with the attenuation rule of the laser-induced shock wave pressure in the materials. The SAED pattern of the top surface (region C) presented halo ring characteristics, which may have been caused by the presence of either very fine grains or amorphous phases. To clarify the microstructural morphology, HRTEM observations were carried out, as shown in Figure 5b, which confirmed the amorphous structure of the material for 10 nm in thickness. For depths greater than 10 nm, the microstructure was composed of both nanocrystalline and amorphous phases. This was also confirmed by the corresponding SAED pattern of region C, in which both halo rings and diffraction spots were presented.

### 3.2. Surface Nanocrystallization

The results described in Section 3.1 show that different microstructures were generated after LSP treatment with different laser parameters, including high-density dislocations, dislocation tangles, dislocation cells, and nano-grains. Hence, the surface nanocrystallization process was similar to the one caused by conventional severe plastic deformation methods, such as shot peening and surface mechanical attrition treatment [3,6]. On the other hand, there are many differences in the surface nanocrystallization mechanism between LSP and conventional shot peening.

**Figure 6:**
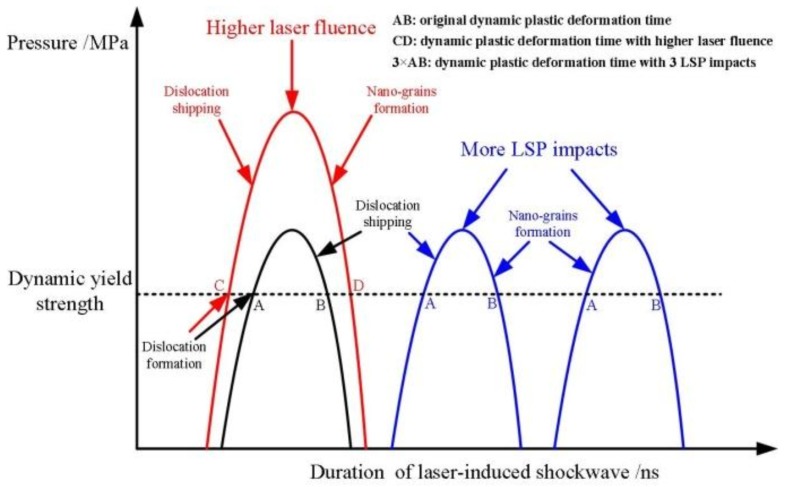
The principle of the action of the shockwave for increasing the laser power density or LSP impact. *t*_AB_ and *t*_CD_ are the plastic deformation times at a fixed power density and at a higher power density, respectively; 3-AB is the dynamic plastic deformation time with the three impacts.

In conventional shot peening, the plastic deformation with a strain-rate of about 10^3^ s^−1^ occurs in the contact region when the material surface is struck by hard particles, and the surface nanocrystallization is a stepwise evolution process. As for LSP, when the shock wave pressure induced by LSP was larger than the material dynamic yield strength, plastic deformation occurred and the strain-rate of the plastic deformation reached 10^6^ s^−1^. During the plastic deformation process, the atoms were forced to move because of the laser-induced shock wave. According to the homogeneous nucleation theory [30,31], the atoms usually moved in arrays at the shock wavefront, which led to the formation of dislocations. Unlike the dislocation formation process during conventional shot peening, these dislocations were generated at the shock wavefront in the direction of the shock wave propagation. If dislocations were generated, the subsequent shock wave caused the dislocations to ship; if the dislocations met each other or other crystal defects, the dislocation slipping ceased. The dislocations, therefore, gather in correspondence of special locations, where there is a great resistance to their movement, giving rise to the formation of two special statuses: dislocation tangles and dislocation cells. The process of microstructural change was completed extremely rapidly, which was attributed to the fact that the action of the shock wave lasted for only a few tens of nanoseconds. As shown in Figure 4 and in the previous work [29], the surface nanocrystallization was realized when the laser power density or the LSP impacts increased. An increase in the laser power density or LSP impact corresponds to an increase in the laser energy injection into the materials, or an increase in the dynamic plastic deformation time of the laser-induced shock wave, as described in Figure 6. The injected laser energy was transformed into material plasticity energy by the action of the shock wave and was stored in the crystal defects such as dislocations and grain boundaries. The degree of grain refinement increases with the laser energy injected in the material. Therefore, when the dynamic plastic deformation time of the shock wave increases, the dislocations were further driven to slip. Lastly, the dislocation cells transformed into nano-grains, while the dislocation walls transformed into nano-grain boundaries by the annihilation and rearrangement of dislocations. On the other hand, due to the local temperature rising during the LSP process (detailed discussion in Section 3.3), the dynamic recrystallization took place. Therefore, the surface nanocrystallization mechanism consisted of three main steps: (i) the formation of high-density dislocations; (ii) the pileup of dislocation to dislocation cells; (iii) the formation of sub-grain boundaries and of surface nanocrystalline artifacts through continuous energy injection and dynamic recrystallization.

### 3.3. Mechanism of Laser-Induced Amorphization

Amorphization is considered as an extreme case of grain refinement. There are two methods for synthesizing amorphous structures in metallic materials [32,33]: (i) ‘‘freezing’’ the dynamic disorder of a liquid using an extremely high cooling rate, that is, rapid quenching; (ii) destabilizing and ‘‘melting’’ a solid to the amorphous state by introducing chemical and/or structural disorder, which is known as solid-state amorphization. For example, Ye et al. [27] found that an amorphous phase could be generated by LSP and discussed the formation mechanism based on the plasticity theory. Similar results and formation mechanisms were reported by Wang et al. [34], who found that the threshold peak pressure for amorphization of a NiTi alloy was about 3.3 GPa (the pressure is 4.4 GPa in this case). Meyers [35,36] found that shear bands were generated in AISI 304 stainless steel and germanium under shock loading conditions, and the formation of an amorphous phase was observed in these shear bands. The liquid-solid structure induced by the local temperature rising and subsequent fast cooling were the main reasons for the formation of an amorphous phase; however, the LSP-induced surface amorphization of titanium alloys has not been reported in the literature so far. Therefore, it is necessary to investigate the mechanism of surface amorphization by LSP on titanium alloys.

According to the results reported in References [34,35,36,37], the crystal-to-amorphous transition can be attributed to the local temperature rising to the melting point of the material due to plastic deformation and its subsequent fast cooling. Worswick and Yang [38] assumed that 5% of the plastic deformation work was stored in the grain defects and 95% was transformed into heat. In addition, the duration of the plastic deformation induced by LSP was only a few tens of nanoseconds. Thus, this thermodynamic process at the shock wavefront was regarded as an adiabatic process. The adiabatic temperature rising during the course of the dynamic loading could be calculated as [39] follows:

(1) $$\Delta T=\frac{\beta }{\rho C_{p}}\int_{0}^{\varepsilon_{f}}\sigma d\varepsilon$$

where *β* is the Taylor–Quinney coefficient which characterizes the portion of plastic deformation work converted into heat (assumed to be 0.9 in this case), *ρ* is the density (4.48 g/cm^3^ for the present TC11 titanium alloy), *C_p_* is the specific heat capacity (0.48 J/(g∙K) for the present TC11 titanium alloy), *σ* is the flow stress, and *ε_f_* is the true strain in the final state. The dynamic mechanical behavior in the plastic deformation process is usually described by the Johnson–Cook constitutive model and the flow stress *σ* induced by LSP is expressed by Equation (2) [40]. Thus, the temperature rising could be expressed by Equation (3) after substituting the Johnson–Cook constitutive equation.

(2) $$\sigma =(\sigma_{0}+B\varepsilon^{n})\left(1+C\ln\frac{\dot{\varepsilon }}{\dot{\varepsilon_{0}}}\right)$$

(3) $$\Delta T=\frac{0.9(1+C\ln\dot{\varepsilon }/\dot{\varepsilon_{0}})}{\rho C_{p}}(\sigma_{0}\varepsilon_{f}+\frac{B\varepsilon_{f}{}^{n+1}}{n+1})$$

where *σ*_0_ and $\dot{\varepsilon_{0}}$ are the initial field strength and reference strain-rate. *B*, *C*, and *n* are the constants of the Johnson–Cook constitutive equation. For the titanium alloys, the relative constants are as follows: *σ*_0_ is 1030 MPa (shown in Table 2), $\dot{\varepsilon_{0}}$ is 10^−2^/s, and $\dot{\varepsilon }$ is 10^7^/s; *B*, *C*, and *n* are 1092 MPa, 0.014, and 0.93, respectively [41]; and *ε_f_* is about 2 for the local strain. The local temperature rising ∆*T* was found to be about 1990 K, which was higher than the melting temperature *T_m_* (about 1800 K) of the TC11 titanium alloy.

During the LSP process, once the temperature in the material reached *T_m_*, melting occurred without consuming any enthalpy of fusion. The temperature rising in the melting region transited into the neighboring regions to the melting point, until the heat flow from the melting zone was no longer sufficient to raise the temperature of its neighbor to *T_m_*. The cooling rate in the melting region was at least of the order of 10^7^ K/s [42], which resulted in the liquid-to-amorphous phase transformation. This is why a 10 nm-thick of amorphous phase was observed by HRTEM in the present TC11 titanium alloy.

### 3.4. Microhardness Distribution

To better characterize the base material behavior to LSP, changes in the properties were evaluated via Vickers microhardness measurements. In this study, the microhardness of samples was tested by the MVS-1000JMT2 microhardness tester (BAHENS, Shanghai, China), using an indentation load of 500 g with a dwell time of 15 s at the section. For each depth of the specimen, the hardness value was regarded as an average of 5 measurement results and a confidence interval of 95%.

**Figure 7:**
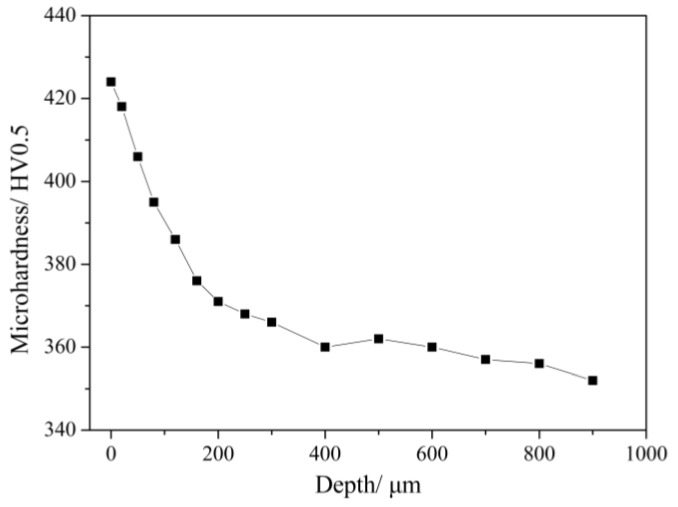
The cross-sectional microhardness of the TC11 titanium alloy with LSP treatment.

The cross-sectional microhardness curves of TC11 titanium alloy after LSP treatment is shown in Figure 7. It is observed that the average value of surface microhardness of the original specimen is approximately 351 HV0.5. After LSP treatment, the microhardness was effectively increased and the maximum value was located at the surface at 424 HV0.5. The affected depth is about 500 μm and the gradient change of the microhardness is consistent with the attenuation of the laser-induced shock wave pressure. The microhardness improvement is attributed to the surface work hardening and microstructural changes induced by LSP. The relationship between microhardness and microstructure can be expressed by the Hall–Petch equation [43]:

(4) $$H_{v}=H_{0}+kd^{-1/2}+\alpha Gb\rho^{1/2}$$

where *H_v_* is the microhardness of the material, *H*_0_ is the original hardness of the material, *k* and *α* are material constants, *d* is the grain size, *G* is the shear modulus of the material, *b* is the Burgers vector, and *ρ* is the dislocation density. After multiple LSP treatments, the grain sizes in the surface layer are refined and high-density dislocation is found in the substrate layer. According to the Hall–Petch model, the microhardness increases after LSP treatment. Due to the thickness being only 10 nm, the effect of the surface amorphousness on microhardness cannot be assessed.

## 4. Conclusions

In this paper, the surface and cross-sectional microstructures of the TC11 titanium alloy were characterized via transmission electron microscopy and high-resolution transmission electron microscopy. According to the different microstructural features, the mechanisms of surface nanocrystallization and amorphization after LSP treatment were discussed. The main conclusions obtained in this work may be summarized as follows:

(1) Surface nanocrystallization was induced by LSP on the TC11 titanium alloy. In the LSP process, the dislocations were generated at the shock wavefront. They then developed into dislocation cells and finally formed the nano-grains by dislocation, slipping under the continuous shock wave, and dynamic recrystallization.
(2) More specially, an amorphous layer of about 10 nm thickness was generated on the top surface above the nanostructured layer. The local temperature rising during the LSP process resulted from the dynamic compression and ultrahigh strain-rate plastic deformation under the laser-induced high-pressure shock wave. The combined effect of the temperature rising to the melting point and the fast cooling caused the surface amorphization of the TC11 titanium alloy.

## Figures and Tables

**Table 1:** The chemical composition of the TC11 titanium alloy.

Composition	Al	Mo	Cr	Zr	Si	Fe	Sn	Ti
Percentage (wt %)	5.8–7.0	2.8–3.8	–	0.8–2.0	0.15–0.40	0.2–0.7	–	Bal

**Table 2:** The static tensile properties of the TC11 titanium alloy at room temperature.

Materials	Yield Strength σ_0.2_ (MPa)	Ultimate Tensile Strength σ_b_ (MPa)	Elongation Rate δ (%)
TC11 titanium alloy	930	1030	9
